# Differences in Body Composition and Lower Limb Strength Between Novice and Amateur Marathon Runners: A Cross-Sectional Study

**DOI:** 10.3390/sports13090287

**Published:** 2025-08-26

**Authors:** Tianxin Shi, Qingzhao Shi, Shuang Ren, Xiaorui Huang, Jun Ren, Xin Gao, Jingxian Zhu

**Affiliations:** 1 Department of Sports Medicine, Peking University Third Hospital, Institute of Sports Medicine of Peking University, Beijing Key Laboratory of Sports Injuries, Engineering Research Center of Sports Trauma Treatment Technology and Devices, Ministry of Education, Beijing 100191, China; shitianxin1234@163.com (T.S.);; 2 School of Kinesiology and Health, Capital University of Physical Education and Sports, Beijing 100191, China

**Keywords:** amateur marathon runners, novice runners, body composition, lower limb strength

## Abstract

This study compared the body composition and strength of the lower extremity parameters between novice runners (NRs) and amateur marathon runners (AMRs). A total of 50 NRs (33.84 ± 4.32 years) and 50 AMRs (33.36 ± 5.55 years) were analyzed cross-sectionally. Bioelectrical impedance analysis measured body composition parameters, and isokinetic testing assessed knee muscle strength. The results showed that compared to AMRs, NRs had lower fat-free mass (FFM), skeletal muscle mass (SMM), and total body water (TBW) (−15, −12, and −5%; all *p* < 0.01) but higher body fat percentage (PBF) and visceral fat area (VFA) (+27 and +32%; both *p* < 0.01). They also had 6% lower knee extensor (KE) strength and 31% lower knee flexor (KF) strength on the dominant legs (DLs) and 14% lower KF strength on the non-dominant legs (NDLs). In addition, their hamstring–quadriceps ratio (H: Q) was 24% lower on the DLs and 9% lower on the NDLs. The NRs exhibited significant negative correlations between PBF, VFA, and knee muscle strength (r = −0.54 to −0.42, *p* < 0.01), while the AMRs had significant negative correlations only for PBF (r = −0.59 to −0.57, *p* < 0.001). In conclusion, the NRs exhibited lower FFM and TBW, higher PBF and VFA, and reduced muscle strength. In contrast, the AMRs exhibited the opposite pattern. These findings suggest that NRs with elevated body fat (BF) indicators should prioritize fat reduction and performance enhancement, while those with lower muscle mass require targeted programs to increase muscle capacity and joint stability. This approach may advance them toward the level of AMRs. Future studies should adopt longitudinal designs to explore how training interventions influence the physiological adaptations observed in runners at different experience levels.

## 1. Introduction

With the rise in public health awareness, running has become one of the most popular fitness exercises across the world [1]. Within the running community, two distinct groups emerge—novice runners (NRs) and amateur marathon runners (AMRs). NRs are commonly associated with those who run <10 miles per week, have no former experience in running, and never take part in a running competition [2]. On the other hand, AMRs are commonly associated with those who run consistently for more than one year and have run more than 40 km per month or have participated in at least one official marathon [3]. Although running is increasingly popular among the general population, the physiological differences between runners at different experience levels remain unclear. In particular, few studies have compared key physical characteristics—such as body composition and lower limb strength—between NRs and AMRs. Understanding these differences is important for developing individualized training strategies and for monitoring physical development as NRs progress toward higher levels of endurance performance [4,5].

Body composition and lower limb strength are core factors in running performance. Previous studies primarily focused on elite or professional athletes [6,7,8], leading to a limited understanding of amateur-level runners, who in fact constitute the majority of the running population. For example, Herrmann et al. [6] found that body composition was a better predictor of running performance than body mass index (BMI) in long-distance runners; they also found that adiposity was negatively correlated with running speed. In the field of exercise science, prior studies have explained the physiological characteristics of professional athletes [7,8]. For example, Chivate et al. [7] measured the lower limb strength of sprinters with different speeds and found that the faster the sprinters, the stronger their muscle strength. In addition, Stachoń et al. [8] measured the body composition of male runners of different distances and found that, compared with sprinters, middle-distance runners had relatively low subcutaneous fat content, a difference reflecting the distinct body composition between the two types of athletes. Quantifying these traits not only provides important baseline characteristics for the majority of the running population but also enables sports scientists and coaches to develop a clearer picture of the physical attributes associated with different stages of running [9]. This understanding informs the development of appropriate training recommendations and supports individualized nutritional guidance for optimizing body composition [10]. Moreover, establishing benchmarks of different running levels allows for the objective assessment of physiological status and monitoring of adaptations [11].

Given the above considerations, this study aims to quantitatively analyze the body composition and lower limb strength of AMRs and NRs using body composition tests and isokinetic muscle strength tests to clarify the specific differences as well as their extent, which can help to reveal the differences in physiological characteristics of people with different running levels. Specifically, this study addresses the following research questions: (1) variations in body composition parameters between AMRs and NRs; (2) differences in knee extensor and flexor strength between the two groups when normalized to body mass; and (3) elucidating these variations may enhance the understanding of the physiological characteristics associated with different running levels. This information can inform NRs’ individualized decisions regarding training volume and intensity by providing comparative physiological profiles of more experienced AMRs, which in turn is important for developing individualized training approaches and monitoring physical development as NRs advance toward greater endurance capacity.

## 2. Materials and Methods

### 2.1. Study Design

This study adopted a sex-balanced design, with male and female participants in a 1:1 ratio (25 males and 25 females in each group), in order to control for sex-related effects on physiological indicators. A stratified purposive sampling approach was used to recruit participants from local running clubs, fitness centers, and online platforms between February 2024 and December 2024. Participants were divided into two groups, NRs and AMRs, which compared the differences in body composition and lower limb strength parameters. All experiments were performed in the Biomechanics Laboratory, Department of Sports Medicine, Peking University Third Hospital. This study was approved by the Ethics Committee of the Third Hospital of Peking University (project identification Number: M2023268). All participants signed an informed consent form before enrollment.

### 2.2. Participants

**Figure 1:**
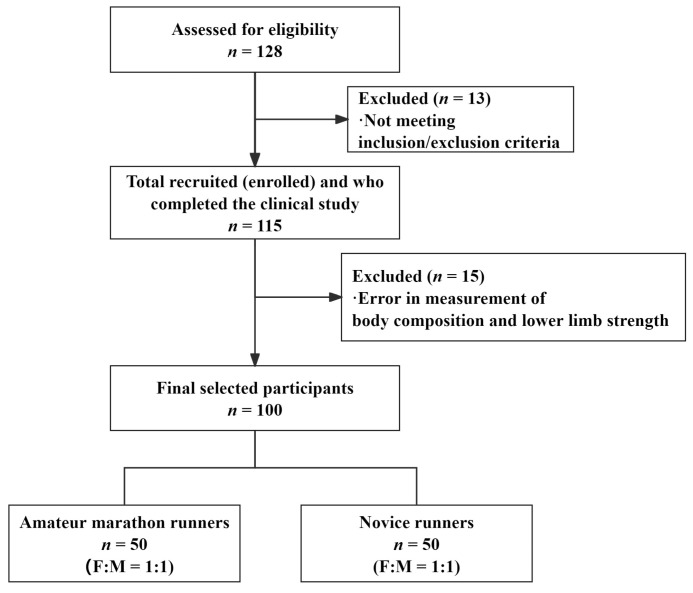
Flow chart of study sample selection. F = female; M = male.

To minimize potential confounding variables, especially those related to sex and age, a matched enrollment strategy was implemented—each participant enrolled in one group was matched with a participant of the same sex and similar age in the other group. Participant selection was strictly based on predefined inclusion and exclusion criteria, not on test outcomes, ensuring the scientific integrity and objectivity of the sampling process. A total of 128 participants were assessed for their eligibility for inclusion in this study. Of the 128 participants, 115 completed the clinical study after excluding individuals who did not meet the inclusion/exclusion criteria. The inclusion criteria were as follows: (1) aged 18–45 years, regardless of sex; (2) not registered with the Chinese Athletic Association; (3) AMRs [3] were described as participants having been running consistently for more than one year and have run more than 40 km per month, or have participated in at least one official marathon; (4) NRs [2] were defined as running <10 miles per week, having no former experience in running, and never taking part in a running competition; and (5) voluntary signed informed consent and commitment to cooperate with the experimental process were provided. The exclusion criteria were as follows: (1) history of lower limb musculoskeletal injuries or surgery; (2) severe cardiovascular, respiratory, or neurological diseases; and (3) inability to comply with experimental requirements or attend tests on time. Among the 115 participants who completed the study, 15 participants were excluded due to errors in the measurements of their body composition and lower limb strength. Consequently, the analytical sample comprised 50 AMRs and 50 NRs (Figure 1). The achieved sample size was larger than the required minimum, thereby providing adequate statistical power. All participants signed an informed consent form before enrollment.

### 2.3. Sample Size Calculation

This study adopted a sex-balanced design, with male and female participants in a 1:1 ratio, in order to control for the sex-related effects on physiological indicators. The a priori sample size was calculated using G*Power 3.1 software based on the effect size (Cohen’s d = 0.65) derived from a pilot study, in which the mean difference between groups was approximately 3.69 units, with a pooled standard deviation of 5.67 units. Using an independent samples *t*-test, with α = 0.05 and power (1-β) = 0.80, a minimum of 34 valid samples per group (total ≥68) was required to detect this effect. To account for a potential dropout rate of 20%, we planned to recruit 50 participants per group (25 males and 25 females), totaling 100 participants. Despite excluding 15 participants due to procedural errors during testing, the final sample size of 100 exceeded the minimum requirement, ensuring sufficient statistical power.

### 2.4. Data Collection

#### 2.4.1. Demographic and Body Composition Data

**Figure 2:**
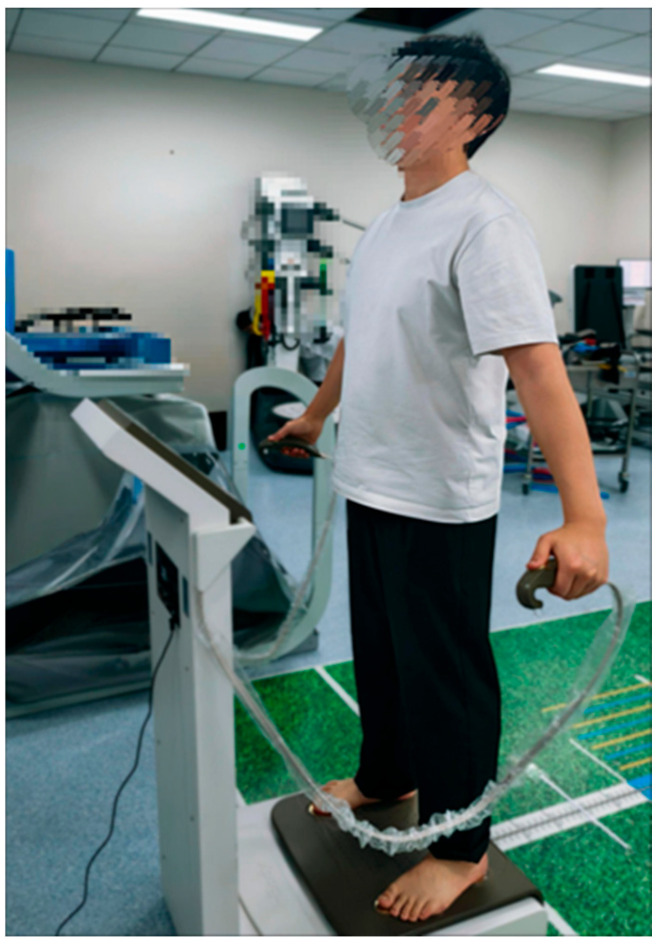
Body composition test picture.

A standardized self-reported form was filled out by all participants, including information about their age, running experience, running volume, sex, height, and dominant leg (DL) [12]; the DL is usually defined as the one that is used to kick a ball. All participants filled in a single measurement of body composition using the bioimpedance device InBody 770 (Biospace, Urbandale, IA, USA). Participants were instructed to fast for 8 h and to avoid strenuous exercise for 24 h before testing. Upon arrival at the laboratory, they were asked to stand barefoot on the platform of the device with the soles of their feet resting on the electrodes, hold the handles with both hands, and remain stationary for 1 min while keeping the elbows fully extended and the shoulder joints abducted by approximately 30° (Figure 2) [13]. The following data were recorded: (1) body fat percentage (PBF, %), which is defined as the proportion of fat mass relative to total body weight, reflecting the ratio of body fat to lean tissue; (2) skeletal muscle mass (SMM, kg), which represents the total amount of skeletal muscle in the body, including associated tissues; (3) fat-free mass (FFM, kg), which comprises the total non-fat tissue in the body, including muscle, bone, organs, and water; (4) total body water (TBW, kg), which is the sum of all water present in the body and constitutes the primary component of fat-free body weight; and (5) visceral fat area (VFA, cm^2^), which refers to the area of fat surrounding the abdominal visceral organs [14]. Among these indicators, previous studies have shown outstanding test–retest reliability (ICC ranging from 0.98 to 0.99 for PBF and from 0.99 to 1.00 for FFM) [15]. Earlier research has also documented a technical measurement error of 4.2% for PBF and 2.4 kg for FFM [16].

This device was used since a previous study reported an almost-perfect correlation using the gold-standard method (dual-energy X-ray absorptiometry, with a Pearson’s correlation coefficient >0.97) and an excellent reliability (ICC > 0.98) [13].

#### 2.4.2. Lower Limb Muscle Strength Features

**Figure 3:**
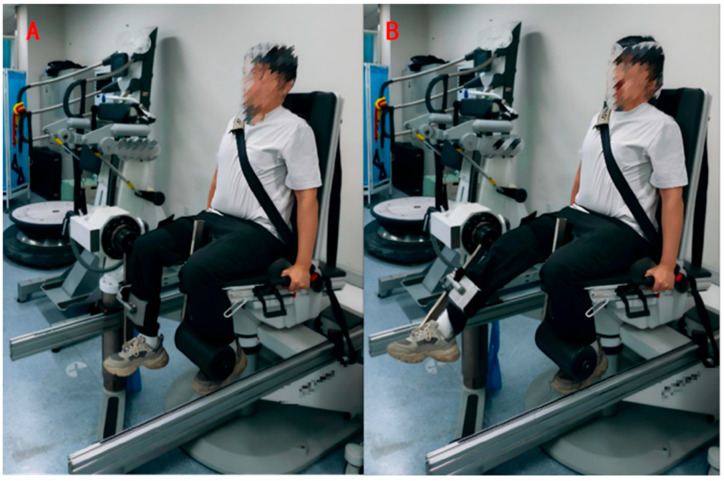
Lower extremity isokinetic strength test pictures: (**A**) initial phase; (**B**) final phase.

Isokinetic muscle strength testing was performed using an isokinetic test evaluation system ( CMV AG; Con-Trex MJ; Physiomed Elektromedizin AG, Schnaittach, Germany). Evidence confirms exceptional reliability (ICC: 0.87–0.98) for this testing methodology [17,18]. The technical error of measurement was reported to range between 6.6 and 10.1% [18]. Before data collection, participants engaged in a 5 min warm-up on a reclining bicycle (Technogym Xt Pro 600 Recline, Cesena, Italy), followed by basic stretching exercises targeting the lower body. Participants were positioned on the dynamometer with 80° hip flexion from anatomical neutral, adhering to manufacturer specifications. The lateral femoral condyle was carefully aligned with the axis of the dynamometer’s power arm. The lever arm length was individualized for each participant, and the resistance pad was placed just proximal to the medial malleolus [19]. To ensure stability, the participant’s torso, pelvis, tested thigh, and non-tested calf were securely immobilized. The calibration angle and starting position were set at a 90° knee flexion (Figure 3A) [20]. The knee’s range of motion was defined from a 10 to 90° flexion (Figure 3B) [21]. Additionally, gravity correction was applied to account for the influence of calf and power arm weights on peak torque during knee extension. Before each formal test, the participant was given a clear safety range of knee motion and was familiarized with the test procedure. After positioning, participants completed 1–2 submaximal familiarization trials. Subsequently, five maximal isokinetic contractions (60°/s) were executed for knee flexion–extension, with a 60 s rest between testing sequences. Testing commenced with the DL, followed by the non-dominant leg (NDL) after a 3 min recovery period. A single laboratory technologist conducted all isokinetic assessments to minimize inter-rater variability. Testing included knee extensors (KEs) and flexors (KFs) bilaterally at a 60°/s angular velocity. It has been shown that the most used angular velocity in force analysis is 60°/s [22]. Jenner et al. [23] found moderate levels of retest reliability for knee flexion and extension movements (ICC = 0.64–0.74) by performing five tests at a mean isokinetic speed of 60°/s on the knee joint while observing effect sizes (ESs) ranging from 0.01 to 0.43 and typical errors (TEs) ranging from 5.65 to 22.87, all of which were characterized by small-to-moderate levels.

Body weight-normalized peak torque (PT/BW) served as the primary outcome measure for KEs and KFs. Additionally, the H:Q ratio was calculated by dividing the mean concentric KF PT by the mean concentric KE PT over the five repetitions [24,25]. The bilateral strength differences for the KFs and KEs were calculated based on the following equation [26,27]:

$$\begin{array}{c} Bilateral\;strength\;deficit=\\ \frac{DL\;\mathrm{peak}\;\mathrm{torque}-NDL\;\mathrm{peak}\;\mathrm{torque}}{DL\;\mathrm{peak}\;\mathrm{torque}}\times 100\;(\%) \end{array}$$

### 2.5. Statistical Analysis

Data are presented as mean ± standard deviation (SD). The Kolmogorov–Smirnov test was used to check the normality of the data [28]. Paired *t*-tests were conducted to evaluate the differences between the DL and NDL performance. Differences in body composition and muscular strength between novice runners and AMRs were assessed using independent-sample *t*-tests. Effect sizes were interpreted according to Cohen’s d thresholds [29]: Cohen’s d < 0.2 (small), 0.2  ≤ Cohen’s d < 0.8 (moderate), and 0.8 ≤ Cohen’s d (large). Associations between knee extensor strength and body composition metrics were examined with Pearson correlations. Correlation magnitudes were classified as small (0.1 ≤ |r| < 0.3), medium (0.3 ≤ |r| < 0.5), or large (0.5 ≤ |r| ≤ 1.0). All computations were executed in IBM SPSS 27.0 (SPSS Inc., Chicago, IL, USA), with statistical significance set at *p* < 0.05.

## 3. Results

### 3.1. Demographic and Anthropometric Characteristics

Table 1 shows that there were no significant differences between the two groups in terms of age, height, weight, BMI, sex distribution, or the DL proportion (all *p* > 0.05). In contrast, running experience and running volume differed substantially between groups (all *p* < 0.05). This balance was not coincidental but was intentionally achieved as part of the study design. Specifically, participants were recruited based on predefined inclusion criteria to ensure comparable distributions in age and sex across the two groups. This matching strategy was implemented to minimize potential confounding effects from non-running-specific variables, thereby enabling a clearer interpretation of group differences in running experience, volume, and physiological outcomes.

### 3.2. Characterization of Body Composition in AMR and NR Groups

Table 2 shows that there were significant differences between the two groups in terms of FFM, SMM, TBW, PBF, and VFA (all *p* < 0.01), and the effect sizes were of practical significance. The AMR group had lower PBF and VFA than the NR group (both *p* < 0.001), with the effect size indicating a substantial magnitude of difference. Conversely, the AMR group had higher FFM, SMM, and TBW (all *p* < 0.01), and the effect sizes for these metrics were of moderate magnitude. Specifically, the AMR group had a 15% higher FFM, a 12% higher SMM, and a 5% higher TBW than the NR group. Meanwhile, the AMR group had a 27% lower PBF and a 32% lower VFA than the NR group.

### 3.3. Characterization of Knee Muscle Strength in AMR and NR Groups

Table 3 shows that statistically significant differences were observed in PT/BW and H:Q between the two groups (all *p* < 0.05). Specifically, compared to the AMR group, the NR group demonstrated a 6% lower KE strength on the DLs, as well as a 31% lower KF strength. On the NDLs, the NR group showed 14% lower KF strength, while no significant difference was found in NDL KE strength between the two groups (*p* > 0.05). Similarly, the H:Q of the NR group was 24 and 9% lower than that of the AMR group on the DLs and NDLs, respectively. In terms of bilateral limb comparison within the AMR group, the PT/BW of the DLs was 4.07 and 15.38% higher than that of the NDLs for KEs and KFs, respectively (both *p* < 0.05). Additionally, the H:Q value of the DLs was 11.79% higher than that of the NDLs (*p* < 0.05). In terms of bilateral limb comparison within the NR group, the PT/BW of the DLs was 3.45 and 3.10% higher than that of the NDLs for KEs and KFs, respectively (both *p* < 0.05).

### 3.4. Correlation Between Knee Muscle Strength and Body Composition in AMR and NR Groups

**Figure 4:**
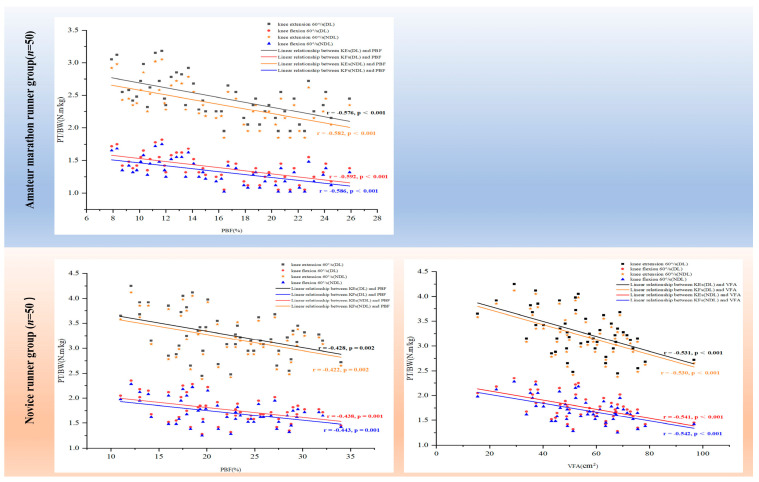
Pearson’s correlation (r) of body composition estimates to isokinetic strength. Note. PBF = body fat percentage; VFA = visceral fat area; DL = dominant leg; NDL = non-dominant leg.

Figure 4 shows that in the novice group, both PBF and VFA exhibited significant negative correlations with knee muscle strength (r = −0.54 to −0.42; *p* < 0.01), whereas in the AMR group, PBF was also significantly negatively correlated with knee muscle strength (r = −0.59 to −0.57; *p* < 0.001); however, VFA did not correlate significantly with knee muscle strength (*p* > 0.05). In addition, FFM, SMM, and TBW were not significantly correlated with knee muscle strength across groups and on the DLs and NDLs (*p* > 0.05).

## 4. Discussion

This cross-sectional study aimed to investigate the differences in body composition and knee muscle strength between novice runners and AMRs, as well as to examine the correlations between these variables, thereby complementing previous research in this area [30,31,32]. The results showed that compared to the AMRs, the NRs had higher PBF and VFA but lower FFM, SMM, and TBW. In terms of lower limb strength, the NRs had lower PT/BW than the AMRs. Furthermore, negative correlations were observed between fat-related indices (PBF and VFA) and knee muscle strength in the NRs, while in the AMRs, only PBF remained significantly correlated with strength.

When comparing the body composition of the NRs and AMRs, the AMRs showed significantly higher FFM, SMM, and TBW, while exhibiting lower PBF and VFA compared to the NRs. This finding was supported by multiple studies [33,34,35,36,37,38,39,40,41]. For example, Micheli et al. [33] conducted whole-body impedance measurements on 893 soccer players and discovered that FFM was lower in athletes with lower performance levels compared to elite athletes. Similarly, Rosado et al. [34] found that ultra-distance runners were characterized by low levels of BF. Elite athletes consistently demonstrated higher SMM and TBW compared to their inexperienced and less-successful counterparts [35,36,37]. It is worth noting that individuals training for a marathon frequently focus only on running and strengthening lower limbs, forgetting about general training and building skeletal muscle mass in the upper limbs and the trunk. Body composition analyses indicated an increased water content in these runners compared to that of athletes, which might be related to their enhanced skeletal muscle mass [38]. Nikolaidis et al. [39] observed that more experienced recreational marathoners (with ≥4 marathons) had a lower adiposity than NRs. Additionally, Clemente-Suarez et al. [40] assessed the body composition of 52 male mountain marathon runners and found that the better-performing runners weighed less and had lower PBF and BF compared to the less-successful runners. Kutac et al. [41] found that runners who ran an average of 21.6–31.4 km/week had a lower VFA compared to those who did not exercise. These studies emphasized the differences in body composition between runners of different distances, especially in endurance sports. Such data provided valuable reference points for practitioners and facilitated more timely adjustments to training plans, nutritional strategies, and recovery protocols [42]. Integrating regular body composition monitoring into training cycles can thus serve as a valuable tool for optimizing physical condition, tracking physiological adaptations, and guiding evidence-based decisions in amateur running contexts.

When comparing the lower limb strength of NRs and AMRs, the AMRs displayed a higher knee muscle strength. An analysis of the PT/BW indicated that the strength differences on the DL and NDL of the KEs and KFs of marathon runners were 4.07 and 15.38%. According to the literature, interlimb asymmetric differences are significant when they reach at least 15% [43], and it can be inferred that strength asymmetry was present in the KFs on both legs of the AMRs. The H:Q serves as a key biomechanical indicator, quantifying the strength balance between agonist and antagonist muscle groups at the knee joint [44]. International studies suggest that a normal H:Q falls between 30 and 90%, while domestic research proposes a 50 to 60% range, considering racial differences among Asians. However, the majority of studies still maintain that the optimal H:Q lies between 50 and 80%, where an overly low (<50%) or excessively high (>80%) ratio could potentially precipitate knee joint dysfunction [45]. At a 60°/s angular velocity, the AMRs demonstrated significantly elevated H:Q ratios versus the NRs on both DLs (*p* < 0.05) and NDLs (*p* < 0.05). This result could be attributed to the fact that the AMRs had significantly higher knee muscle strength on both legs compared to the NRs, thus resulting in a higher H:Q. Notably, the H:Q was higher on the DLs of marathon runners compared to the NDLs (*p* < 0.05), favoring the DL performance. This observation aligned with the findings of Andrade et al. [46], who documented similar muscle strength imbalances between the KFs and KEs in recreational runners. One study highlighted the presence of interlimb asymmetry in long-distance runners and demonstrated that the posterolateral asymmetry of KEs might be linked to the marked posterolateral asymmetry of tibial loading during running [47]. These observations enriched the existing body of knowledge on muscular characteristics in NRs and AMRs, providing useful normative data that could be employed in physical assessments and comparative analyses.

Significant inverse associations between PBF and knee muscle strength in both the NRs and AMRs highlight the detrimental impact of excess adiposity on lower limb strength, a key component of running efficiency. This finding aligns with previous research indicating that a higher fat mass impairs performance in tasks requiring speed and power output [48]. It is especially relevant for NRs, who typically have higher a PBF and lower baseline strength compared to more experienced athletes [49]. From a practical standpoint, these results support incorporating body composition assessment—particularly targeting PBF—into early-stage training programs for NRs. Aerobic exercise and dietary interventions aimed at reducing fat mass, combined with progressive resistance training to increase FFM and SMM, can improve running economy and establish a foundation for safe, long-term performance gains [50]. These findings emphasize the interconnectedness of morphological and functional characteristics, underscoring the importance of comprehensive assessments that include both body composition and muscle strength measures. The current evidence on body composition–knee strength relationships remains nascent. Future investigations should expand to novel morphometric indicators (e.g., phase angle and extracellular-to-total body water ratio) and dynamic strength parameters.

### 4.1. Strengths and Limitations

This study was a comparative analysis of 1:1 matched runners both with and without marathon training experience. Both groups had a 1:1 male-to-female ratio and an average age of 33 years. This rigorous matching minimized confusion between age and sex. Although causal inferences could not be drawn due to the cross-sectional nature of the study, these findings provided important physiological benchmarks that might guide the characterization and monitoring of runners at different experience levels.

However, this study had several limitations. Firstly, the relatively small sample size, despite matching the two groups in a 1:1 ratio, might not have been sufficient to detect small differences. Secondly, measurement errors and individual differences could have affected the results. Even with standardized testing equipment and methodology, some degree of error in measuring muscular strength was possible, and inter-individual differences might have influenced the significance of the findings. Thirdly, it was worth noting that only a single speed of 60°/s was used in the isokinetic test of knee strength, which limited the comprehensive analysis of the change rule of knee strength under different speed conditions to a certain extent. Fourthly, given the cross-sectional design of the study, only correlational analyses were possible, which precludes causal inference. Future research should include more longitudinal studies to further investigate the effects of marathon training on body composition and knee muscle strength. Fifthly, the study conducted only one body composition assessment, limiting data reliability and accuracy validation. Failing to perform two trials for retest reliability data led to deficiencies in the study’s stability and consistency. Future research should increase measurements to better ensure data reliability and support accurate body composition assessment. Finally, it is worth noting that although training experience was quantified in this study, correlation analyses between training experience and body composition parameters were not included. This decision was based on the study’s primary objective to compare groups with distinct training backgrounds rather than to investigate continuous relationships. Future studies with larger samples and more detailed training metrics are encouraged to explore these potential associations.

### 4.2. Practical Applications

From a practical perspective, both body composition and lower limb strength indicators can inform targeted interventions to enhance running performance. NRs with a relatively high PBF or VFA should not only engage in aerobic endurance training but also incorporate complex, multi-joint strength exercises and make dietary adjustments to reduce excess adiposity and improve running economy, while those with a lower SMM or FFM should perform resistance training to enhance muscular capacity and strengthen the active musculoskeletal system for propulsion and joint stability. Additionally, compared to AMRs, NRs show significantly lower KF strength, especially on their DL. They should prioritize KF strengthening exercises such as Nordic hamstring curls and Romanian deadlifts while also improving KE strength through deep squats, lunges, or deadlifts to maintain muscular balance. Furthermore, AMRs exhibit interlimb asymmetry in KF strength, highlighting the importance of unilateral strength training, such as single-leg squats, lunges, or hamstring curls, to restore balance and prevent overuse injuries.

## 5. Conclusions

In conclusion, significant differences in body composition and lower limb strength exist between NRs and AMRs. The AMRs had a higher FFM and TBW while exhibiting a lower PBF and VFA and stronger knee muscles, with interlimb strength asymmetries in their KFs; however, no significant intralimb strength asymmetries were found. These findings suggest that NRs with elevated body fat (BF) indicators should prioritize fat reduction and performance enhancement, while those with lower muscle mass require targeted programs to increase muscle capacity and joint stability. This approach may advance them toward the level of AMRs. Future studies should adopt longitudinal designs to explore how training interventions influence the physiological adaptations observed in runners at different experience levels.

## Figures and Tables

**Table 1:** Descriptive statistics for demographic and anthropometric characteristics.

	AMR Group (*n* = 50)	NR Group (*n* = 50)	*p*	Cohen’s d
Age (years)	33.36 ± 5.55	33.84 ± 4.32	0.74	0.10
Running experience (years)	4.34 ± 2.00 ***	0.54 ± 0.21	<0.001	1.42
Running volume (km/month)	66.02 ± 12.75 ***	19.48 ± 6.62	<0.001	4.58
Height (cm)	171.32 ± 6.14	170.48 ± 8.51	0.69	0.11
Weight (kg)	64.51 ± 7.06	66.49 ± 11.35	0.46	0.21
BMI (kg/m^2^)	20.95 ± 1.77	21.75 ± 2.56	0.21	0.36
Sex (male/female)	25/25	25/25	1.00	0.00
DL (right/left)	40/10	45/5	0.169	0.14

Note. BMI = body mass index; DL = dominant leg; AMR = amateur marathon runner; NR = novice runner. ***: *p* < 0.001.

**Table 2:** Descriptive statistics for body composition in the two groups.

	AMR Group (*n* = 50)	NR Group (*n* = 50)	*p*	Cohen’s d
FFM (kg)	49.97 ± 7.64 **	45.40 ± 8.41	0.005	0.57
SMM (kg)	28.06 ± 4.61 **	25.13 ± 5.07	0.003	0.60
TBW (kg)	36.94 ± 5.38 **	33.21 ± 5.95	0.001	0.66
PBF (%)	16.13 ± 5.07 ***	22.17 ± 5.78	<0.001	1.11
VFA (cm^2^)	37.17 ± 11.38 ***	54.93 ± 15.65	<0.001	1.30

Note. FFM = fat-free mass; SMM = skeletal muscle mass; TBW = total body water; PBF = body fat percentage; VFA = visceral fat area; AMR = amateur marathon runner; NR = novice runner. **: *p* < 0.01; ***: *p* < 0.001.

**Table 3:** Descriptive statistics for knee muscle strength in the two groups.

		AMR Group (*n* = 50)		NR Group (*n* = 50)		P_PT/BM_	Cohen’s d_PT/BM_	P_H:Q_	Cohen’s d_H:Q_
		PT/BM (N.m/kg)	H:Q (%)	PT/BM (N.m/kg)	H:Q (%)				
KEs	DL	2.46 ± 0.32^ab^	68.70 ± 12.08^ab^	2.32 ± 0.33^b^	55.60 ± 11.68	0.034	0.43	<0.001	1.10
	NDL	2.36 ± 0.31	60.60 ± 11.64^a^	2.24 ± 0.31	55.80 ± 11.47	0.056	0.39	0.040	0.42
	*p*	<0.001	<0.001	0.008	0.848				
	Cohen’s d	0.32	0.68	0.25	0.02				
KFs	DL	1.69 ± 0.20^ab^		1.29 ± 0.20^b^		<0.001	2.00		
	NDL	1.43 ± 0.20^a^		1.25 ± 0.19		<0.001	0.92		
	*p*	<0.001		0.027					
	Cohen’s d	1.30		0.21					

Note. KEs = knee extensors; KFs = knee flexors; DL = dominant leg; NDL = non-dominant leg; °/s = degrees per second; PT/BM = peak torque/body mass; N.m/kg = Newton meter/kilogram; AMR = amateur marathon runner; NR = novice runner; H:Q = hamstrings–quadriceps ratio. a: A significant difference compared to the novice group, *p* < 0.05. b: A significant difference compared to the non-dominant leg, *p* < 0.05.

## Data Availability

The datasets generated and analyzed in the current study are not available publicly, but are available upon reasonable request from the corresponding author.

## Abbreviations

The following abbreviations are used in this manuscript:

AMR	Amateur marathon runner
NR	Novice runner
KEs	Knee extensors
KFs	Knee flexors
DL	Dominant leg
NDL	Non-dominant leg
SD	Standard deviation
FFM	Fat-free mass
SMM	Skeletal muscle mass
TBW	Total body water
PBF	Body fat percentage
VFA	Visceral fat area
BF	Body fat
BMI	Body mass index
H: Q	Hamstrings: Quadriceps ratio
